# Feasibility of On-Site CT-FFR Analysis in Ruling Out In-Stent Restenosis on Cardiac PCCT

**DOI:** 10.3390/jcdd13070308

**Published:** 2026-07-05

**Authors:** Isabelle Ayx, Felix Waßmer, Lena Lichti, Matthias F. Froelich, Sylvia Buettner, Theano Papavassiliu, Stefan O. Schoenberg, Thomas Germann

**Affiliations:** 1 Department of Radiology and Nuclear Medicine, University Medical Center Mannheim, Heidelberg University, Theodor-Kutzer-Ufer 1-3, 68167 Mannheim, Germany; isabelle.ayx@medma.uni-heidelberg.de (I.A.); felix.wassmer@umm.de (F.W.); lena.lichti@stud.uni-heidelberg.de (L.L.); matthias.froelich@medma.uni-heidelberg.de (M.F.F.); stefan.schoenberg@umm.de (S.O.S.); 2 Department of Medical Statistics, Biomathematics and Information Processing, University Medical Center Mannheim, Heidelberg University, Theodor-Kutzer-Ufer 1-3, 68167 Mannheim, Germany; sylvia.buettner@medma.uni-heidelberg.de; 3 First Department of Medicine-Cardiology, University Medical Centre Mannheim, Theodor-Kutzer-Ufer 1-3, 68167 Mannheim, Germany; theano.papavassiliu@umm.de

**Keywords:** photon-counting computed tomography, coronary artery disease, CT-fractional flow reserve, in-stent restenosis

## Abstract

The evaluation of stents in coronary computed tomography angiography (CCTA) is still a major topic in cardiovascular imaging. Using Photon-Counting Detector CT (PCCT) may improve the assessment of coronary stents and make on-site CT-FFR analysis feasible for ruling out in-stent restenosis (ISR). In this study, patients with previous coronary stent implantation who underwent CCTA using PCCT and subsequent invasive catheter angiography (ICA) were included. Stent characteristics such as location and length were reported. CT-FFR measurements were taken 1.8 cm before and after the stent, with a value of ≤0.80 defined as hemodynamically significant under respecting the diagnostic accuracy drop in the gray zone between 0.76 and 0.80. Delta CT-FFR with a cut-off value of ≥0.06, indicating hemodynamic significance, was determined. Any ISR and interventional treatment during the following ICA was recorded. Diagnostic performance metrics, including sensitivity, specificity, positive predictive value (PPV), and negative predictive value (NPV), were calculated for post-stent CT-FFR and Delta CT-FFR in detecting ISR. Patients were followed up to evaluate the rate of major adverse cardiovascular events (MACE) 6 months after CCTA. A total of 19 patients (5 female, 14 male, median age 69 years) were enrolled in this study. In most cases, coronary stents were located in the proximal LAD with a median stent length of 70.2 mm. Pathological CT-FFR < 0.76 distal to the stent was detected in 6 cases (31.6%), while pathological Delta CT-FFR ≥ 0.06 occurred in 14 cases (73.7%). ICA was performed in three of these patients, with ISR confirmed in two cases. These findings yield sensitivity and NPV of 100% for both post-stent CT-FFR and Delta CT-FFR for excluding ISR with a superior specificity (76.5% vs. 29.4%) and overall diagnostic accuracy (78.9% vs. 36.8%) for post-stent CT-FFR. Two patients reported a myocardial infarction in follow-up; however, neither of them was located in the territory of the stented coronary artery. This study outlines the feasibility of on-site CT-FFR analysis using PCCT in excluding ISR in coronary stents with a high diagnostic accuracy. These findings highlight the need to extend the benefits of CT-FFR analysis for non-invasive assessment of possible ISR regarding personalized risk stratification and therapy planning.

## 1. Introduction

Cardiovascular diseases (CVD) represent the leading global cause of mortality [1], with a projected increase in crude mortality rates over the coming decades due to worldwide population aging [2]. Consequently, cardiac imaging is increasingly critical for the early diagnosis and subsequent management of relevant CVDs to mitigate long-term sequelae [3]. The clinical value of cardiac computed tomography (CT) as a non-invasive alternative to invasive coronary angiography (ICA) in the diagnostic algorithm for coronary artery disease (CAD) has been firmly established in recent years [4]. This paradigm shift is further substantiated by the DISCHARGE trial, which demonstrated that in patients with stable chest pain, an initial CT imaging strategy is associated with fewer procedure-related complications compared to initial ICA. Moreover, major adverse cardiovascular event (MACE) rates remained comparable between the two strategies, as coronary computed tomography angiography (CCTA) effectively prevented unnecessary invasive procedures [5].

Currently, cardiac CT is well established for the exclusion of CAD and the risk stratification for MACE. However, it exhibits intrinsic limitations in reliably determining the hemodynamic significance of heavily calcified, high-grade coronary stenoses [6,7].

To overcome this anatomical limitation, CT-derived fractional flow reserve (CT-FFR) was developed as a post-processing adjunct to CCTA. CT-FFR enables the non-invasive assessment of the physiological relevance of coronary lesions, yielding precise functional data without requiring additional invasive procedures [8,9]. Clinical evidence demonstrates that CT-FFR surpasses standalone CCTA in identifying hemodynamically significant stenoses and serves as an independent prognostic indicator for survival and cardiac events [10]. Traditionally, initial CT-FFR models utilized complex, three-dimensional (3D) reconstructions and computational fluid dynamics (CFD), necessitating off-site processing at dedicated core laboratories. To address these logistical bottlenecks, on-site methodologies—incorporating reduced-order models and machine learning—were developed to optimize computational efficiency and time. These rapid approaches are now well-validated in contemporary research [11,12,13,14]. The integration of CT-FFR into clinical workflows substantially reduces the necessity for diagnostic ICA and fosters individualized, patient-centered therapeutic planning [15]. Consequently, these functional imaging techniques have been formally incorporated into recent international clinical practice guidelines [16]. Furthermore, the implementation of photon-counting CT scanners (PCCT) represents a transformative advancement in cardiac diagnostics, driving cutting-edge research in software-guided CT-FFR and comprehensive plaque characterization [17]. The superior spatial and temporal resolution of PCCT, combined with an enhanced signal-to-noise ratio, markedly reduces beam-hardening artifacts. This ultimately enables accurate and robust evaluation of coronary arteries and stents, even in the presence of severe calcification [18,19].

Phantom studies utilizing PCCT have yielded promising results for coronary stent evaluation, demonstrating superior in-stent lumen visibility and quantitative image characteristics compared to conventional energy-integrating detector (EID) systems [20,21]. Initial in-human evaluations also suggest that the non-invasive assessment of coronary stent patency via PCCT is highly feasible, providing robust image quality [22]. Given the frequent incidence of in-stent restenosis (ISR) in patients undergoing percutaneous coronary intervention [23,24] and the limited use of invasive fractional flow reserve (FFR) for post-procedural surveillance due to associated complications and costs [25], CT-FFR presents a compelling alternative for patient management. The synergistic integration of on-site CT-FFR with PCCT has been shown to be effective in identifying hemodynamically significant coronary artery stenoses, potentially reducing the reliance on purely diagnostic ICA [16]. Although these diagnostic benefits have been primarily observed in patients without prior cardiovascular stents, accurately quantifying potential ISR or novel stent-related stenosis remains a critical imperative in cardiovascular imaging, despite a current lack of robust clinical validation. Currently, no commercially available, regulatory-approved CT-FFR solutions exist for stented segments. Metal artifacts, altered intracoronary anatomy and persistent hemodynamic conditions present technical challenges that must be addressed to ensure reliable assessments of stented segments.

To extend the utility of these non-invasive diagnostic advancements to patients with previously implanted cardiac stents, this retrospective study aims to evaluate the presence of ISR and its hemodynamic significance. Specifically, we will apply on-site CT-FFR software to PCCT datasets and correlate these findings with invasive re-catheterization results and the incidence of MACE.

## 2. Materials and Methods

### 2.1. Study Design and Patient Population

**Figure 1:**
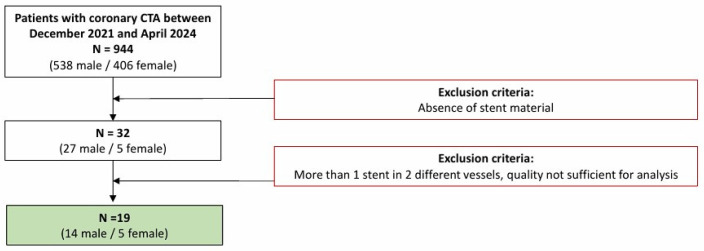
Flowchart for patient enrollment within the study, indicating exclusion and inclusion criteria.

Patients who received a clinically indicated cardiac CT following the current ESC guidelines [26] using the novel PCCT between December 2021 and February 2024 were enrolled in this study. Inclusion criteria were defined by the presence of stents in the coronary arteries. Patients were excluded in case of severe image artifacts due to motion or foreign bodies, and if exceeding stents in more than one coronary artery. In addition, clinical parameters were gathered retrospectively through a specialized questionnaire regarding common CVD risk factors. Based on inclusion and exclusion criteria, 19 patients (14 male, 5 female, median age 69 years, range 43–82 years) were enrolled in this study (Figure 1). The study was conducted following the principles of the Declaration of Helsinki and obtained approval from both the institutional review board and the local ethics committee (ID 2021-659).

### 2.2. Cardiac CT Image Acquisition

All 19 patients were examined using the first-generation whole-body dual-source PCCT system (NAEOTOM Alpha; Siemens Healthcare GmbH, Forchheim, Germany). The scan protocol employed a prospective electrocardiogram (ECG)-gated sequential mode with a tube voltage of 120 kV and automatic dose modulation. The CARE keV BQ setting was set to 64, and the gantry rotation time was configured to 0.25 s. To keep heart rates below 65 beats per minute and enhance image quality through a vasodilatory effect, patients were first administered intravenous β-blocker (5–15 mg), followed by 0.8 mg glyceryl trinitrate in the form of a sublingual spray, unless contraindicated. A non-enhanced cardiac CT scan was initially performed to assess coronary artery calcification and calculate the Agatston Score using an axial scan with 3 mm slice thickness, 3 mm increment, and a Qr36 kernel, processed with dedicated software (syngo.via Software, Version VB60, Siemens Healthcare GmbH, Forchheim, Germany). This was followed by a contrast-enhanced scan using 80 mL of iodine contrast agent (Imeron 350, Bracco Imaging Deutschland GmbH, Konstanz, Germany) and a 20 mL saline chaser (NaCl 0.9%) administered at a flow rate of 5–6 mL/s based on patient weight. The CCTA was triggered by bolus tracking in the ascending thoracic aorta.

### 2.3. CCTA and CT-FFR Analysis

**Figure 2:**
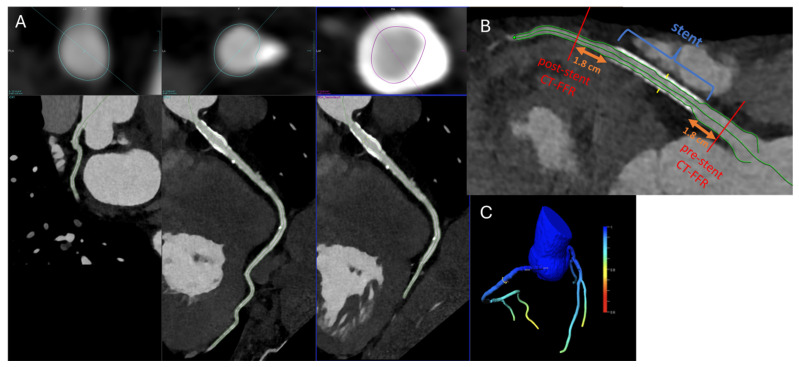
CT-FFR analysis workflow in a 64-year-old male with a proximal right coronary artery (RCA) stent. (**A**) Axial view of the stented RCA segment (Segment 1) showing contrast-enhanced lumen (blue/pink) and curved multiplanar reconstruction with green path markers. (**B**) Curved multiplanar reconstructions displaying detailed CT-FFR measurement points. (**C**) Color-coded 3D CT-FFR model illustrating flow values along the RCA, with red indicating regions of reduced fractional flow reserve.

Stent characteristics such as location, length, and ISR were reported. In this context, stent length was defined as total length of the stented vessel segment where multiple overlapping stents were used. CCTA was evaluated for stenosis or occlusions based on the current CAD-RADS 2.0 criteria. For CT-FFR analysis, contrast-enhanced axial reconstructed images with a slice thickness of 0.6 mm, an increment of 0.4 mm, a matrix size of 512 × 512, a Bv40 soft tissue kernel, and quantum iterative reconstruction level 3 were chosen. CT-FFR analysis was done using dedicated on-site software by creating a 3D model of the coronary vessel tree to first visualize the anatomic course of each vessel (syngo.via, Siemens Healthcare GmbH, Forchheim, Germany). The vessel lumen was visualized in a longitudinal section with detailed imaging. For each stent, the center of the stent within the respective vessel was marked. This reference point provided the CT-FFR value within the stent. To ensure reproducibility in the CT-FFR measurements before and after the stent, additional markers were placed. Due to the fact that the maximum pressure drop occurs within the stenosis, while maximum pressure recovery is reached 0.8–1.7 mm distal of the stenosis [27,28] the markers were placed 1.8 cm proximal and 1.8 cm distal to the stent’s start and end, with CT-FFR values calculated for each. In case there were several consecutive stents within a vessel the distance between them was measured. If the distance was below 1.8 cm from each other those were considered as one and measured with 1.8 cm respectively before the first and after the last stent. The CT-FFR values across the vessel were then displayed in the overview image using these markers. An example CT-FFR analysis workflow is shown in Figure 2. A value of ≤0.80 was defined as potentially hemodynamically significant, as recommended in the literature [29]. Additionally, however, we added a borderline CT FFR value of 0.76–0.80 to respect the drop in diagnostic accuracy as outlined in the literature for a more detailed analysis [30]. For the CT-FFR analysis, the Delta CT-FFR was determined by subtracting the pre-stent value from the post-stent value with a threshold of ≥0.06 for hemodynamic significance [31]. To show the relationship between Delta CT-FFR and stent length, the ratio Delta CT-FFR/stent length × 100 was determined.

### 2.4. ICA and Follow-Up Analysis

ICA, when indicated, was performed according to the current clinical guidelines [32]. For each patient, the degree of ISR was estimated by at least 1 of 6 senior cardiologists. Any interventional treatment, such as balloon angioplasty, repeat (drug-eluting) stent implantation, or other procedures, was recorded.

Six months after the initial CT scan, all patients were contacted via phone. For each patient, the occurrence of MACE, including myocardial infarction, nonfatal stroke, or cardiovascular death, was recorded. Furthermore, any requirement for additional hospitalization due to heart failure or unstable angina pectoris was documented.

### 2.5. Statistical Analysis

All statistical analyses were performed using SAS software (Version 9.04, SAS Institute Inc., Cary, NC, USA). A two-sided significance level of α = 0.05 was defined to determine statistical significance. Descriptive statistics were used to summarize baseline patient characteristics, with continuous variables reported as mean ± standard deviation or median with interquartile range, as appropriate. The Shapiro–Wilk test was applied to assess the normality of distribution for continuous variables within each subgroup.

Diagnostic performance metrics, including sensitivity, specificity, positive predictive value (PPV), and negative predictive value (NPV), were calculated for both post-stent CT-FFR and Delta CT-FFR in detecting ISR, as partially confirmed by ICA. Comparative analyses between groups (e.g., normal, borderline, and pathological CT-FFR values) were conducted using the non-parametric Mann–Whitney U test or Kruskal–Wallis test for continuous variables and the chi-square (χ^2^) test or Fisher’s exact test for categorical variables, as appropriate.

To assess the association between CT-FFR or Delta CT-FFR values and clinical endpoints, including MACE, ICA, and ISR rates, univariate analyses were performed. Odds ratios (ORs) with corresponding 95% confidence intervals (CIs) were calculated to estimate the relative risk of undergoing ICA or having ISR based on categorized CT-FFR and Delta CT-FFR cut-off values (<0.75 and ≥0.06, respectively). Where applicable, receiver operating characteristic (ROC) curve analysis was used to evaluate the discriminatory power of CT-FFR metrics, and area under the curve (AUC) values were reported.

## 3. Results

### 3.1. Patient Population

Patient characteristics of the 19 included patients are outlined in Table 1. The overall median age was 69 years (range of 43–82 years) with 5 female (26.3%) and 14 male (73.7%) patients. Due to the presence of coronary stents, the Agatston calcium score was not calculated in any patient; however, in at least three cases, coronary arteries without visible calcifications were identified.

In most cases, the LAD was treated with a stent (n = 13, 68.4%). The majority of these were located in the proximal or mid LAD (n = 11, 84.6%), although in two cases, two stents were placed consecutively. Two LAD stents (15.4%) were located in the distal portion of the LAD (segment 8). In four cases (21.1%), the stent was located in the RCA, three of which were in the proximal segment of the vessel and one in the distal segment. The remaining two stents were located in the posterolateral artery of the LCX and the intermediary branch (10.5%). The median stent length was 70.2 mm with a range of 30.0 to 174.6 mm.

### 3.2. CTA Evaluation

In 17 of 19 cases, stenosis was detected in the non-stented coronary arteries. Eight of these patients had non-significant stenosis of <50% (CAD-RADS 1/S, n = 6, 31.6% and CAD-RADS 2/S, n = 3, 15.8%, respectively). One patient showed moderate stenosis of 50–69% (CAD-RADS 3/S, 5.3%), and seven patients had severe stenosis of >70% in at least one stent-free coronary artery, resulting in a CAD-RADS 4A/S (36.8%). In the remaining two patients, no stenosis was detected in the coronary arteries, corresponding to CAD-RADS 0/S. In none of the evaluated cases was an ISR or occlusion found.

### 3.3. CT FFR Analysis

The median CT-FFR proximal to the stent was 0.99 with a range of 0.9 to 1.0, which means that no pathological values were measured in any of the cases. The median CT-FFR within the stent was 0.89 (0.62–1.0) with one borderline reduced value of 0.77 in the intermediary branch and one decreased CT-FFR of 0.62 within a stent of the LAD. CT-FFR distal to the stent shows a median of 0.81, with a wide range from 0.22 to 0.98. In 10 cases (52.6%), the value was within the normal range, while three cases (15.8%) had a borderline CT-FFR of 0.78 to 0.79. In 6 of 19 cases (31.6%), a pathological CT-FFR distal to the stent was detected with values < 0.76 (0.22–0.73). Delta CT-FFR was within the normal range in five patients (26.3%), while a pathological Delta CT-FFR ≥ 0.06 was detected in 14 cases (73.7%). The median Delta CT-FFR/stent length × 100 of all patients was 1.69, with a range of 0.22 to 8.87. Low ratios were detected in patients without pathological CT-FFR > 0.8 distal of the stent (0.22–1.98) and in patients with borderline CT-FFR of 0.76–0.8 distal of the stent (0.90–1.82). In contrast, patients with pathological CT-FFR < 0.75 distal to the stent had significantly higher ratios of 6.26 on average (1.26–8.87).

**Figure 3:**
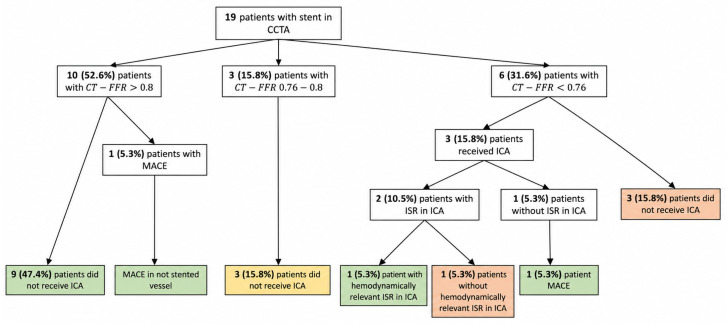
Overview of diagnostic performance of post-stent CT-FFR.

Post-stent CT-FFR analysis classified 6 of 19 patients (31.6%) as having pathological values (<0.75), 3 patients (15.8%) as having borderline values (0.75–0.79), and 10 patients (52.6%) as having normal values (≥0.80). None of the patients with normal or borderline CT-FFR values underwent ICA. Among the six patients with pathological post-stent CT-FFR values, ICA was performed in three cases (50%), and in two of these (33.3%), ISR was confirmed. One of the ISR cases required PCI due to hemodynamic significance. Based on these findings, post-stent CT-FFR demonstrated a sensitivity of 100%, specificity of 76.5%, PPV of 33.3%, NPV of 100%, and an overall diagnostic accuracy of 78.9% for the detection of ISR confirmed by ICA (Figure 3).

**Figure 4:**
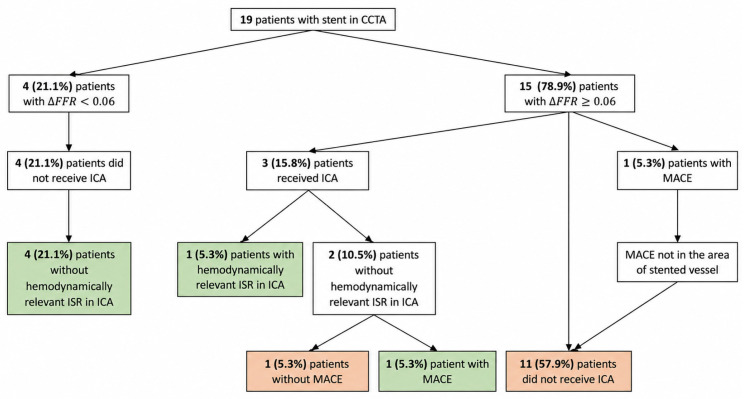
Overview of diagnostic performance of Delta CT-FFR.

In contrast, Delta CT-FFR analysis (defined as the difference in CT-FFR values proximal vs. distal to the stent) identified 14 patients (73.4%) with pathological values (≥0.06) and 5 patients (26.3%) with normal findings (<0.06). As with post-stent CT-FFR, none of the patients with normal Delta CT-FFR underwent ICA. ICA was performed in three patients with pathological Delta CT-FFR, with ISR confirmed in two cases. For Delta CT-FFR, this yielded a sensitivity of 100%, but with a markedly lower specificity of 29.4%, PPV of 14.3%, NPV of 100%, and a diagnostic accuracy of 36.8% for ISR detection (Figure 4).

These findings highlight the high sensitivity and excellent NPV of both post-stent CT-FFR and Delta CT-FFR for excluding ISR, while suggesting superior specificity and overall diagnostic accuracy for post-stent CT-FFR (Table 2).

### 3.4. ICA and Follow-Up Analysis

In three patients (15.8%), an ICA was performed during follow-up. IRS was detected in two of these patients (89.5%), with subsequent PCI being performed in one of these two cases. In the remaining cases (84.2%), no further intervention was done after CCTA.

**Figure 5:**
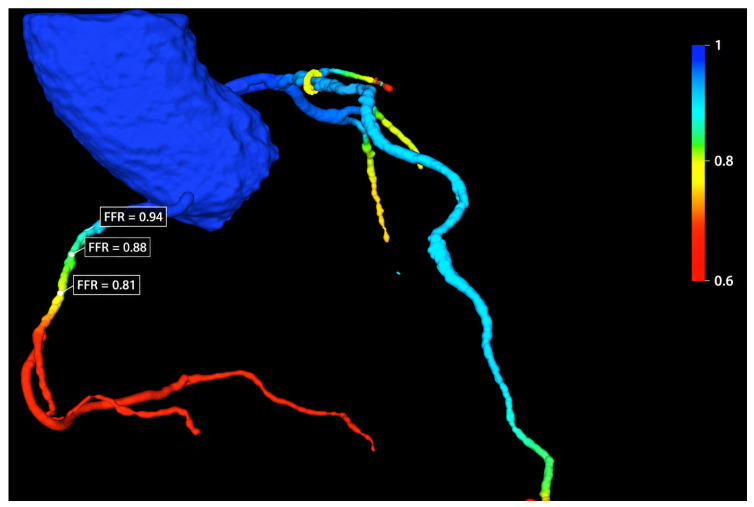
A 75-year-old female with a proximal LAD stent (Segment 6). Physiological post-stent CT-FFR (0.92; Delta FFR 0.03) was achieved while pathological CT-FFR in the RCA (70–99% stenosis, CAD-RADS 4A) preceded before posterior-wall MI. LAD remained free of significant stenosis on ICA.

A total of 18 patients were successfully contacted to query the MACE criteria. No cardiovascular events were identified in 16 of the patients surveyed (89.9%). Two patients reported a myocardial infarction; however, each infarction was not located in the supply territory of the stented vessel (Figure 5). One patient could not be reached for follow-up.

## 4. Discussion

To our knowledge, this is the first evaluation of in-stent CT-FFR using photon-counting CT, whose improved spatial resolution and reduced blooming mitigate the in-stent artifacts that have limited conventional coronary CT. Given the novelty of this setting, we evaluated two candidate post-stent metrics—the absolute post-stent CT-FFR and the Delta CT-FFR—without presupposing their relative performance. The absolute metric showed good diagnostic accuracy (78.9%), whereas Delta CT-FFR performed poorly (accuracy 36.8%). This divergence is mechanistically expected. Coronary perfusion pressure declines continuously along the epicardial vessel even without a focal stenosis, a fall that is accentuated by diffuse atherosclerosis: in angiographically normal vessels of patients with coronary disease FFR averages ~0.89, and even truly normal arteries may reach 0.92 [33,34]. A Delta CT-FFR of 0.06 may thus reflect physiological or diffuse tapering rather than focal impairment—which might be even more severe through stent implantation, so that a pathological Delta can coexist with a fully preserved distal value—a constellation not conventionally regarded as significant [35]. We therefore regard the absolute post-stent CT-FFR as the more robust metric in this context and consider Delta CT-FFR exploratory and complementary rather than standalone; a residual focal gradient may nonetheless carry prognostic relevance, as low post-procedural FFR predicts adverse vessel-related events even when the angiographic result appears optimal [36,37].

Most recently, machine learning (ML) applications, using artificial intelligence tools, have been developed to calculate CT-FFR, which may quickly analyze large amounts of data with the ability of learning automatically and adapting to new inputs. In the assessment of the coronary arteries, these applications yield a high diagnostic performance, comparable to that obtained from computational fluid dynamics, as well as invasive FFR [38]. Conversely, the role of CT-FFR, even when performed with ML algorithms, remains unclear in patients with prior coronary stenting.

Tang et al. recently investigated the feasibility and prognostic value of ML-based CT-FFR in predicting cardiovascular adverse events in patients with prior stent implantation [39]. To validate the use of ML-based CT-FFR, they retrospectively selected 33 patients from the CHINA CT-FFR study with a coronary stent, an invasive FFR assessment, and follow-up CTA at least 3 months after the procedure. They reported a good correlation of CT-FFR with invasive FFR, and an accuracy of 86% to detect hemodynamically significant ISR. Exploring the role of ML-based CT-FFR in predicting MACE in 115 patients with coronary stents, they indicated age and follow-up Delta CT-FFR/length as the only two variables independently associated with MACE at follow-up. However, given the high number of ISR < 50% in CCTA, the question remains as to the significance of CT-FFR in patients at high risk for future MACEs. A recent study by Xu et al. evaluated the diagnostic accuracy of subtraction CT-FFR using deep learning reconstruction (DLR) versus hybrid iterative reconstruction (HIR) in patients with heavily calcified coronary lesions or prior stent implantation [40]. Subtraction CT-FFR DLR showed excellent diagnostic performance, with a sensitivity and NPV of 100% for both calcified-related stenoses and stent evaluations. Specificity and PPV were 71.4% and 63.0% for calcifications, and 83.3% and 88.9% for stents, respectively, resulting in an overall accuracy of 80.8% and 92.9%. While subtraction CT-FFR DLR outperformed HIR, the difference was not statistically significant (*p* > 0.05). These findings highlight the high diagnostic reliability of subtraction CT-FFR, particularly when using deep learning techniques, in complex coronary assessments. In comparison to our study, no PCCT technology was used, and no MACE rate was detected, but the feasibility of CT-FFR analysis in patients with stents was determined as well.

Nevertheless, it remains unclear which cut-off value should be used for assessing CT-FFR. While a value of 0.75–0.80 has been validated for ICA to identify hemodynamically and prognostically significant CAD, the extent to which this threshold applies to CT-FFR remains uncertain. For CT-FFR analysis, a value of <0.8 and a Delta of ≥0.06 is considered hemodynamically significant [29,30]. Matsumura-Nakano et al. found a moderate correlation with invasive FFR, with a significant overestimation of hemodynamic significance, and identified a range of 0.71–0.80, where further ICA should be considered [41]. However, different CT-FFR models were used for evaluation (ML-based vs. CFD), and no distinction was made between native vessel and in-stent lesions. In our study, Delta CT-FFR, when compared with post-stent CT-FFR, was found to overestimate the hemodynamic relevance of coronary lesions with stents, leading to a significantly increased classification of pathological findings. Consequently, both the specificity and overall diagnostic accuracy of Delta CT-FFR values appear lower when directly compared to post-stent CT-FFR. This highlights the importance of employing a multiparametric evaluation approach when interpreting Delta CT-FFR results, especially given the altered hemodynamics in stented vessels; an adaptation of the diagnostic cut-off values for Delta CT-FFR may be required to ensure accurate evaluation.

Furthermore, the diagnostic accuracy of CT-FFR can be substantially compromised by metallic blooming artifacts or severe calcifications, which can artificially reduce the perceived luminal diameter and lead to falsely pathological FFR values [42]. In one of our cases, a prominent diagonal branch of the LAD artery originated directly at the distal stent edge. This geometric configuration created an abrupt, anatomically discontinuous reduction in the vessel diameter immediately post-stent, which significantly skewed the CT-FFR evaluation. Because natural vessel tapering is inherently absent within stented segments, the sudden caliber change distal to the stent is magnified. This abrupt transition can lead to an overestimation of the lesion’s hemodynamic significance. Therefore, in patients with coronary stents, precise characterization of the local stented vessel geometry is mandatory for the accurate interpretation of CT-FFR results. The reported diagnostic performance metrics for CT-FFR and Delta CT-FFR should be interpreted with caution because the reference standard ICA was not systematically applied to the entire study population. This introduces a potential verification bias that may artificially inflate sensitivity and NPVs. Moreover, only three patients underwent ICA and ISR was confirmed in only two cases. Consequently, the calculated diagnostic performance measures are based on a very limited number of angiographically verified events and should therefore be regarded as exploratory rather than definitive. Occult ISR in patients who did not undergo ICA cannot be completely excluded. Despite these limitations, this study provides preliminary evidence regarding the feasibility of on-site CT-FFR assessment in stented vessels.

In conclusion, to our knowledge, this is the first study to establish the feasibility of on-site CT-FFR for excluding ISR in stented patients using PCCT. Because conventional commercial platforms routinely exclude this demographic—despite cardiovascular disease remaining the leading global cause of mortality—extending CT-FFR capabilities to patients with coronary stents is critically necessary to optimize personalized risk stratification and therapeutic planning.

## Figures and Tables

**Table 1:** Systematic overview of patient characteristics (LAD = left anterior descending artery, LCX = circumflex branch, RCA = right coronary artery). *p*-values represent Shapiro–Wilk tests for normality and do not indicate between-group comparisons.

Parameter	Result
Sample size (N)	19
Age (median, range)	69 years (43–82 years)
Sex female male	5 (26.32%) 14 (73.68%)
Stent location (vessel) LAD Intermediate branch LCX RCA	13 (68.42%) 1 (5.26%) 1 (5.26%) 4 (21.05%)
Stent position within the vessel proximal mid segment distal	14 (73.68%) 1 (5.26%) 4 (21.05%)
Mean stent length (mean, range)	70.2 mm (30.0–174.6 mm)
CAD-RADS classification grade 0 grade 1 grade 2 grade 3 grade 4A	2 (10.53%) 6 (31.58%) 3 (15.79%) 1 (5.26%) 7 (36.84%)

**Table 2:** Diagnostic parameters of Delta CT-FFR versus CT-FFR in patients with coronary stents (based on a combined clinical–radiological reference standard with ICA, CCTA and follow-up). Borderline findings were classified as non-pathological).

Parameter	Delta CT-FFR	CT-FFR
Sensitivity	100%	100%
Specificity	29.4%	76.5%
Positive Predictive Value	14.3%	33.3%
Negative Predictive Value	100%	100%
Diagnostic Accuracy	36.8%	78.9%

## Data Availability

The data presented in this study are available on request from the corresponding author.

## Abbreviations

The following abbreviations are used in this manuscript:

AUC	Area under the curve
CAD	Coronary artery disease
CAD-RADS	Coronary artery disease reporting and data system
CCTA	Coronary computed tomography angiography
CI	Confidence interval
CT	Computed tomography
CT-FFR	Computed tomography-fractional flow reserve
CVD	Cardiovascular disease
DLR	Deep learning reconstruction
ECG	Electrocardiogram
EID	Energy-integrating detector
ESC	European Society of Cardiology
HIR	Hybrid iterative reconstruction
ICA	Invasive catheter angiography
ISR	In-stent restenosis
LAD	Left anterior descending artery
LCX	Circumflex branch
MACE	Major adverse cardiovascular event
ML	Machine learning
NPV	Negative predictive value
OR	Odds ratio
PCCT	Photon-Counting computed tomography
PCI	Percutaneous coronary intervention
PPV	Positive predictive value
RCA	Right coronary artery
ROC	Receiver operating characteristic
